# Supplementary material for: Development and validation of interpretable machine learning models for triage patients admitted to the intensive care unit
Source: PLoS One. 2025 Feb 18;20(2):e0317819. doi: 10.1371/journal.pone.0317819 (PMC11835250; doi:10.1371/journal.pone.0317819)
Supplement: S1 Text — (PDF) [file pone.0317819.s001.pdf]

S1 Text

Primary Code Used in This Study

Code for Outputting Model Performance Metrics (Example: Gradient Boosting)

```
import pandas as pd
import numpy as np
from sklearn.model_selection import train_test_split
from sklearn.preprocessing import StandardScaler
from sklearn.ensemble import GradientBoostingClassifier
from sklearn.metrics import (
    f1_score, precision_score, recall_score, classification_report,
    roc_auc_score, precision_recall_curve, auc, confusion_matrix,
    brier_score_loss
)
from imblearn.under_sampling import RandomUnderSampler
from sklearn.calibration import calibration_curve
import matplotlib.pyplot as plt

# Load data
data = pd.read_csv(r'D:\ml\data1.csv')    # Ensure the correct file path and name

# Define predictors and target variable
predictors = data.columns.difference(['hadm_id', 'sepsis', 'icu_los', 'outcome', 'death', 'icu'])
X = data[predictors]
y = data['icu']

# Apply RandomUnderSampler for class balancing
rus = RandomUnderSampler(random_state=42)
X_resampled, y_resampled = rus.fit_resample(X, y)

# Split data into training and test sets
X_train, X_test, y_train, y_test = train_test_split(X_resampled, y_resampled, test_size=0.2,
random_state=42)

# Standardize features
scaler = StandardScaler()
X_train_scaled = scaler.fit_transform(X_train)
X_test_scaled = scaler.transform(X_test)

# Train Gradient Boosting Classifier
model = GradientBoostingClassifier(n_estimators=100, learning_rate=0.1, random_state=42)
model.fit(X_train_scaled, y_train)
```

```
# Make predictions and evaluate
y_pred = model.predict(X_test_scaled)
y_scores = model.predict_proba(X_test_scaled)[:, 1]

# Precision, Recall, F1-Score
precision = precision_score(y_test, y_pred)
recall = recall_score(y_test, y_pred)
f1 = f1_score(y_test, y_pred)
print(f"\nPrecision: {precision:.4f}")
print(f"Recall: {recall:.4f}")
print(f"F1 Score: {f1:.4f}")

# Classification report
print("\nClassification Report:")
print(classification_report(y_test, y_pred))

# PR Curve and AUC
precision, recall, _ = precision_recall_curve(y_test, y_scores)
pr_auc = auc(recall, precision)
print(f"\nAUC-PR: {pr_auc:.4f}")

# Confusion matrix with PPV and NPV
tn, fp, fn, tp = confusion_matrix(y_test, y_pred).ravel()
ppv = tp / (tp + fp) if (tp + fp) > 0 else 0
npv = tn / (tn + fn) if (tn + fn) > 0 else 0
print(f"Positive Predictive Value (PPV): {ppv:.4f}")
print(f"Negative Predictive Value (NPV): {npv:.4f}")

# Calibration curve
prob_true, prob_pred = calibration_curve(y_test, y_scores, n_bins=10)
plt.figure()
plt.plot(prob_pred, prob_true, marker='o', label='Gradient Boosting')
plt.plot([0, 1], [0, 1], 'k--', label='Perfectly calibrated')
plt.xlabel('Mean Predicted Probability')
plt.ylabel('Fraction of Positives')
plt.title('Calibration Curve')
plt.legend()
plt.savefig('calibration_curve_gradient_boosting.pdf')
plt.show()

# Brier Score
brier_score = brier_score_loss(y_test, y_scores)
print(f'Brier Score: {brier_score:.4f}')
```

```
# Net benefit at a 5% threshold
threshold = 0.05
y_pred_thresh = (y_scores >= threshold).astype(int)
tn, fp, fn, tp = confusion_matrix(y_test, y_pred_thresh).ravel()
net_benefit = (tp / len(y_test)) - (fp / len(y_test)) * threshold / (1 - threshold)
print(f'Net Benefit at threshold of 5%: {net_benefit:.4f}')
```

ROC Curve Code for Models 2 and 3

```
import pandas as pd
import numpy as np
from sklearn.preprocessing import RobustScaler
from sklearn.model_selection import train_test_split
from sklearn.metrics import roc_curve, auc
import matplotlib.pyplot as plt
from sklearn.linear_model import LogisticRegression
from sklearn.neighbors import KNeighborsClassifier
from sklearn.svm import SVC
from sklearn.naive_bayes import GaussianNB
from sklearn.tree import DecisionTreeClassifier
from sklearn.ensemble import RandomForestClassifier, ExtraTreesClassifier,
GradientBoostingClassifier, AdaBoostClassifier
from imblearn.under_sampling import RandomUnderSampler
from math import sqrt

# Load the dataset
df = pd.read_csv(r'D:\ml\data1.csv')

# Define features (X) and target variable (y)    --model2
predictors = ['temperature', 'heartrate', 'resprate', 'o2sat', 'sbp', 'dbp','pain']
X = data[predictors]
y = data['icu']
# Define features (X) and target variable (y)    --model3
X = df.drop(['hadm_id', 'sepsis', 'icu_los', 'death', 'icu', 'outcome'], axis=1)
y = df['icu']

# Apply RandomUnderSampler for class balancing
rus = RandomUnderSampler(random_state=42)
X_resampled, y_resampled = rus.fit_resample(X, y)

# Normalize features using RobustScaler
X_resampled = RobustScaler().fit_transform(X_resampled)
```

```
# Split the resampled dataset into training and test sets
X_train, X_test, y_train, y_test = train_test_split(X_resampled, y_resampled, test_size=0.2,
random_state=5)

# Define a list of models to evaluate
models = [
    ('Logistic Regression', LogisticRegression(max_iter=1000)),
    ('KNN', KNeighborsClassifier()),
    ('SVM', SVC(kernel='linear', max_iter=1000, C=10, probability=True)),
    ('Naive Bayes', GaussianNB()),
    ('Decision Tree', DecisionTreeClassifier()),
    ('Random Forest', RandomForestClassifier()),
    ('Extra Trees', ExtraTreesClassifier(n_estimators=15, random_state=47)),
    ('Gradient Boosting', GradientBoostingClassifier()),
    ('AdaBoost', AdaBoostClassifier(n_estimators=3))
]

# Prepare the plot
plt.figure(figsize=(10, 8))

# Iterate through each model, train and evaluate
for name, model in models:
    # Fit the model on the training set
    model.fit(X_train, y_train)

    # Predict probabilities for the test set
    y_proba = model.predict_proba(X_test)[:, 1]

    # Compute ROC curve and AUC
    fpr, tpr, thresholds = roc_curve(y_test, y_proba)
    roc_auc = auc(fpr, tpr)

    # Calculate the 95% confidence interval (CI) for AUC using a simplified method
    auc_var = roc_auc * (1 - roc_auc) / len(y_test)
    auc_std = sqrt(auc_var)
    ci_lower = max(0, roc_auc - 1.96 * auc_std)    # Ensure the CI lower boundary is not below 0
    ci_upper = min(1, roc_auc + 1.96 * auc_std)    # Ensure the CI upper boundary is not above 1

    # Plot the ROC curve
    plt.plot(fpr, tpr, lw=2, label='%s (AUC = %0.2f, 95%% CI [%0.2f-%0.2f])' % (name, roc_auc,
ci_lower, ci_upper))

# Add diagonal reference line
plt.plot([0, 1], [0, 1], color='navy', lw=2, linestyle='--')
```

```
# Finalize the plot
plt.xlim([0.0, 1.0])
plt.ylim([0.0, 1.05])
plt.xlabel('False Positive Rate')
plt.ylabel('True Positive Rate')
plt.title('Receiver Operating Characteristic of Various Classifiers')
plt.legend(loc="lower right")

# Save the plot to a file
plt.savefig("roc.pdf")

# Show the plot
plt.show()
```

Code for Plotting DCA (Decision Curve Analysis) Curves

```
import pandas as pd
import numpy as np
from sklearn.model_selection import train_test_split
from sklearn.linear_model import LogisticRegression
from sklearn.naive_bayes import GaussianNB
from sklearn.ensemble import GradientBoostingClassifier, RandomForestClassifier
from sklearn.preprocessing import StandardScaler
from imblearn.under_sampling import RandomUnderSampler
import matplotlib.pyplot as plt

# Load the dataset
data = pd.read_csv(r'D:\ml\data1.csv')    # Ensure the file path and name are correct

# Define predictors and target variable
predictors = data.columns.difference(['hadm_id', 'sepsis', 'icu_los', 'outcome', 'death', 'icu'])

X = data[predictors]
y = data['icu']    # Target variable

# Apply RandomUnderSampler for class balancing
rus = RandomUnderSampler(random_state=42)
X_resampled, y_resampled = rus.fit_resample(X, y)

# Split the dataset into training and test sets
X_train, X_test, y_train, y_test = train_test_split(X_resampled, y_resampled, test_size=0.2,
random_state=42)
```

```
# Scale the features
scaler = StandardScaler()
X_train_scaled = scaler.fit_transform(X_train)
X_test_scaled = scaler.transform(X_test)

# Define models
models = {
    'Logistic Regression': LogisticRegression(max_iter=1000, random_state=42),
    'Naive Bayes': GaussianNB(),
    'Random Forest': RandomForestClassifier(n_estimators=100, random_state=42),
    'Gradient Boosting': GradientBoostingClassifier(n_estimators=100, learning_rate=0.1,
random_state=42)
}

# Define DCA functions
def net_benefit(tp, fp, tn, fn, threshold):
    """Calculate the net benefit for a specific threshold."""
    benefit = tp - fp * (threshold / (1 - threshold))
    return benefit / (tp + fn + fp + tn)

def decision_curve_analysis(y_true, y_prob, thresholds):
    """Perform decision curve analysis and compute net benefits."""
    n = len(y_true)
    y_true = np.array(y_true)
    y_prob = np.array(y_prob)

    net_benefits = []
    for threshold in thresholds:
        tp = np.sum((y_prob >= threshold) & (y_true == 1))
        fp = np.sum((y_prob >= threshold) & (y_true == 0))
        tn = np.sum((y_prob < threshold) & (y_true == 0))
        fn = np.sum((y_prob < threshold) & (y_true == 1))

        nb = net_benefit(tp, fp, tn, fn, threshold)
        net_benefits.append(nb)

    return net_benefits

# Define threshold range
thresholds = np.linspace(0, 1, 100)

# Calculate decision curve analysis for each model
dca_results = {}
for name, model in models.items():
```

```
    model.fit(X_train_scaled, y_train)
    y_pred_proba = model.predict_proba(X_test_scaled)[:, 1]
    nb = decision_curve_analysis(y_test, y_pred_proba, thresholds)
    dca_results[name] = nb

# Create 'Treat None' and 'Treat All' curves
treat_none = np.zeros_like(thresholds)  # Net benefit when treating no patients
treat_all = np.array([np.mean(y_test) - t for t in thresholds])  # Net benefit when treating all patients

# Calculate maximum net benefit for better plot limits
max_net_benefit = max([max(nb) for nb in dca_results.values()] + [max(treat_all)])

# Plot the decision curve analysis
plt.figure(figsize=(10, 8))
colors = ['navy', 'orange', 'green', 'red']
for (name, nb), color in zip(dca_results.items(), colors):
    plt.plot(thresholds, nb, label=name, color=color)
plt.plot(thresholds, treat_none, 'k--', label='Treat None')
plt.plot(thresholds, treat_all, 'k:', label='Treat All')

plt.ylim(-0.035, max_net_benefit + 0.05)
plt.xlabel('Threshold Probability')
plt.ylabel('Net Benefit')
plt.title('Decision Curve Analysis for Different Models')
plt.legend(loc='lower right')
plt.savefig("dca_curve.pdf")
plt.show()
```

Model Interpretation Using SHAP (Example: Gradient Boosting)

```
import pandas as pd
from sklearn.model_selection import train_test_split
from sklearn.ensemble import GradientBoostingClassifier
from imblearn.under_sampling import RandomUnderSampler
import shap
import matplotlib.pyplot as plt
import numpy as np

# Load the dataset
data = pd.read_csv(r'D:\ml\data1.csv')  # Ensure the file path and name are correct

# Define the feature variables
predictors = data.columns.difference(['hadm_id', 'sepsis', 'icu_los', 'outcome', 'death', 'icu']).tolist()
```

```
X = data[predictors]    # Features
y = data['icu']    # Target variable (ICU)

# Apply undersampling to balance the dataset
rus = RandomUnderSampler(random_state=42)
X_resampled, y_resampled = rus.fit_resample(X, y)

# Split the dataset into training and testing sets
X_train, X_test, y_train, y_test = train_test_split(X_resampled, y_resampled, test_size=0.2,
random_state=42)

# Train a Gradient Boosting model
model = GradientBoostingClassifier(random_state=42)
model.fit(X_train, y_train)

# Use `predict_proba` to calculate ICU probabilities
probs = model.predict_proba(X_test)

# Select a specific sample and calculate its ICU probability
sample_index = 16    # Index of the selected sample
icu_probability = probs[sample_index][1]    # Get ICU probability for the 16th test sample

# Print the probability
print(f"Sample {sample_index} ICU Probability (GB): {icu_probability:.4f}")

# SHAP explanation to interpret the model
explainer = shap.Explainer(model, X_train)
shap_values = explainer(X_test)

# Generate a SHAP waterfall plot for the selected sample
plt.figure()
shap.plots.waterfall(shap_values[sample_index], show=False)
plt.title(f"Sample {sample_index} ICU Probability (GB): {icu_probability:.4f}")
plt.savefig('shap_waterfall_plot_gb.pdf', bbox_inches='tight')    # Save the plot as a PDF file
plt.show()
```
