# Supplementary material for: Development and validation of interpretable machine learning models for triage patients admitted to the intensive care unit
Source: PLoS One. 2025 Feb 18;20(2):e0317819. doi: 10.1371/journal.pone.0317819 (PMC11835250; doi:10.1371/journal.pone.0317819)
Supplement: S1 Table — (PDF) [file pone.0317819.s002.pdf]

S1 Table

S1 Table. Performance comparison of ML model (LR) before and after resampling

**Model 2**	Class	Support	Precision	Sensitivity	F1-Score	AUC
Before resampling	Minority class	2008	0.52	0.41	0.42	0.72
	Majority class	35826	0.91	0.88	0.90	
SMOTE	Minority class	35825	0.69	0.65	0.65	0.74
	Majority class	35825	0.67	0.71	0.69	
SMOTE Tomek	Minority class	35676	0.70	0.64	0.64	0.74
	Majority class	35676	0.67	0.71	0.69	
RandomUnderSampler	Minority class	1004	0.68	0.63	0.65	0.74
	Majority class	2023	0.66	0.70	0.68	
**Model 3**						
Before resampling	Minority class	2007	0.59	0.46	0.53	0.79
	Majority class	35826	0.93	0.90	0.92	
SMOTE	Minority class	35825	0.73	0.72	0.73	0.81
	Majority class	35825	0.74	0.73	0.74	
SMOTE Tomek	Minority class	35769	0.73	0.75	0.74	0.82
	Majority class	35769	0.73	0.74	0.74	
RandomUnderSampler	Minority class	1994	0.73	0.70	0.72	0.81
	Majority class	2023	0.74	0.72	0.74	

ML, machine learning; LR, Logistic Regression; AUC, area under the receiver operating characteristic curve.
