# Supplementary figures and images for: Development and validation of interpretable machine learning models for triage patients admitted to the intensive care unit
Source: PLoS One. 2025 Feb 18;20(2):e0317819. doi: 10.1371/journal.pone.0317819 (PMC11835250; doi:10.1371/journal.pone.0317819)

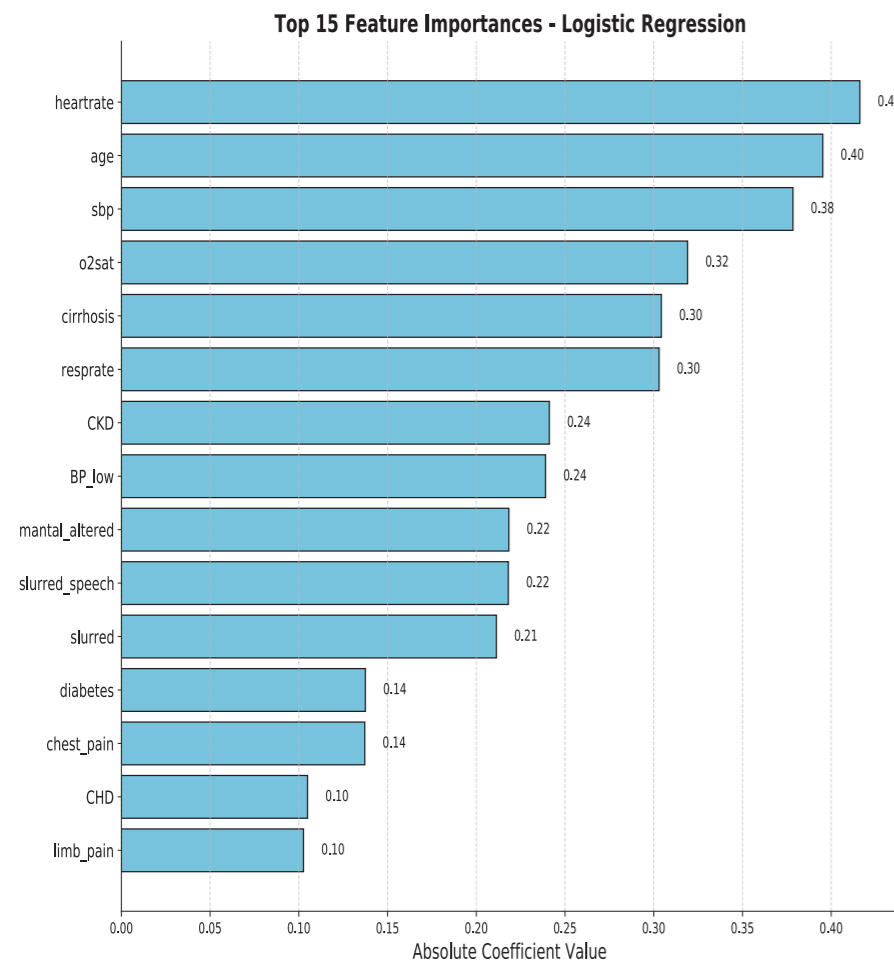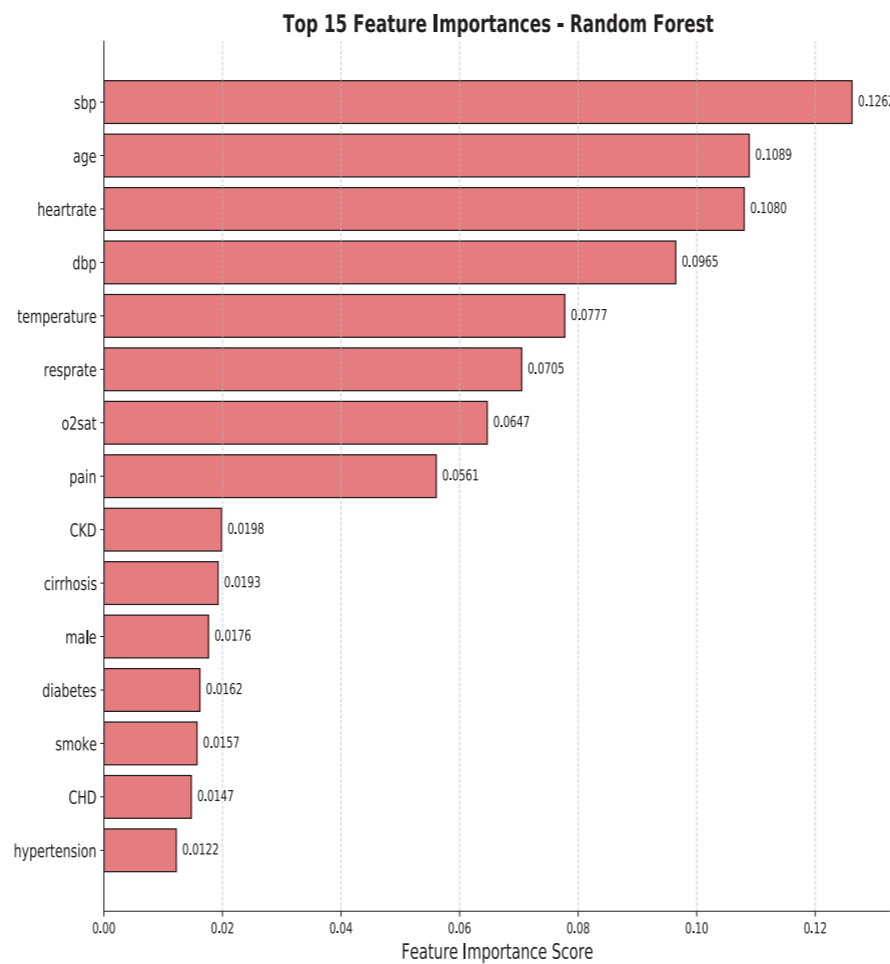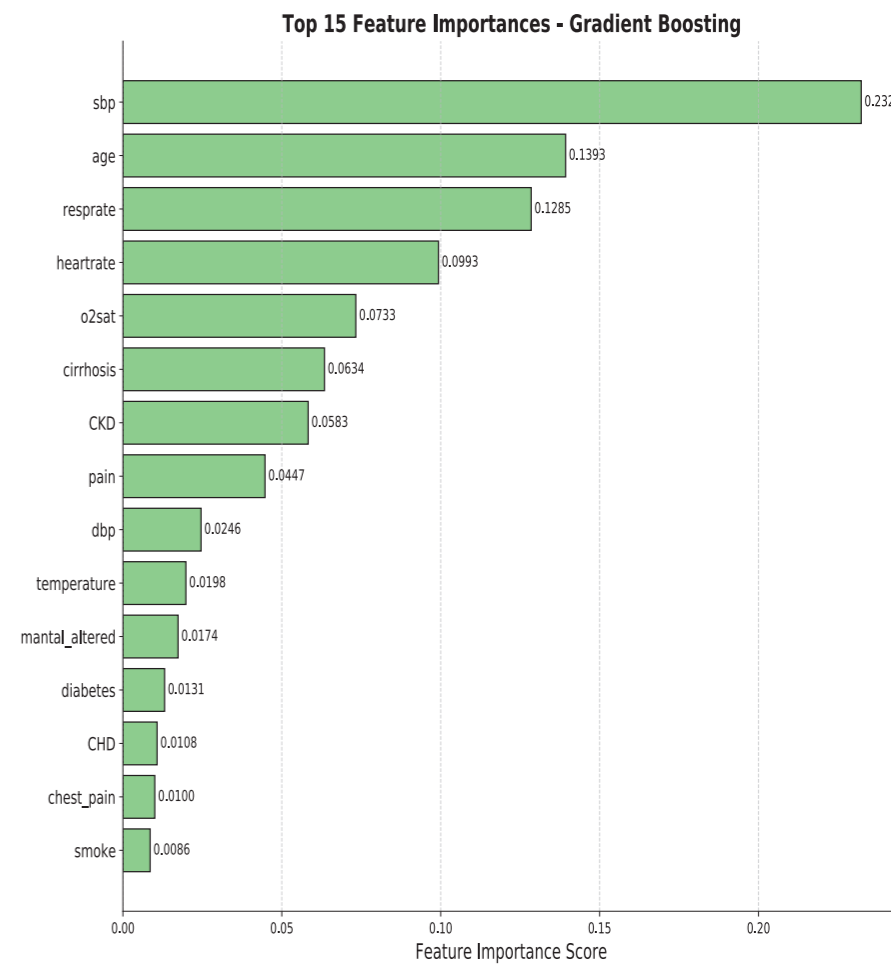

Supplement: S1 Fig — (PDF) [file pone.0317819.s003.pdf]
